# Carpal Tunnel Symptoms With Normal Nerve Conduction Study Findings in Patients With Bifid Median Nerve Treated With Ultrasound-Guided 5% Dextrose Hydrodissection: A Case Series

**DOI:** 10.7759/cureus.36669

**Published:** 2023-03-25

**Authors:** Gamze G Güleç, İlknur Aktaş

**Affiliations:** 1 Physical Medicine and Rehabilitation, Kastamonu Research and Training Hospital, Kastamonu, TUR; 2 Physical Medicine and Rehabilitation, Fatih Sultan Mehmet Research and Training Hospital, Istanbul, TUR

**Keywords:** ultrasound guided interventional pain management, ultrasound imaging, injection, carpal tunnel syndrome, hydrodissection

## Abstract

The most common entrapment neuropathy is carpal tunnel syndrome (CTS), which is caused by compression of the median nerve as it travels through the carpal tunnel in the wrist. Nerve conduction studies (NCS) and ultrasound were used to diagnose CTS but neither method is 100% accurate. The benefit of perineural dextrose injection has been supported in the literature. This article presents three cases with bifid median nerve (BMN) in whom median nerve entrapments were not detected with NCS, and symptom relief was provided with hydrodissection with 2 ml 5% dextrose.

## Introduction

The most common entrapment neuropathy is carpal tunnel syndrome (CTS), which is caused by the compression of the median nerve as it travels through the carpal tunnel in the wrist. Pain, paresthesia, and, less commonly, weakness in the median nerve distribution are common symptoms. Although ultrasound (US) could be used to diagnose CTS, nerve conduction studies (NCS) are the gold standard screening tools for confirming the diagnosis [1]. However, neither method is 100% accurate [1]. Local corticosteroids, dextrose, or platelet-rich plasma injections are used as conservative treatment options [2]. The benefit of perineural dextrose injection has been supported in the literature [3]. In this article, symptom relief with hydrodissection with 2 ml 5% dextrose is presented in three cases with bifid median nerve (BMN) in whom median nerve entrapments were not detected with the NCS.

## Case presentation

Case 1

**Figure 1:**
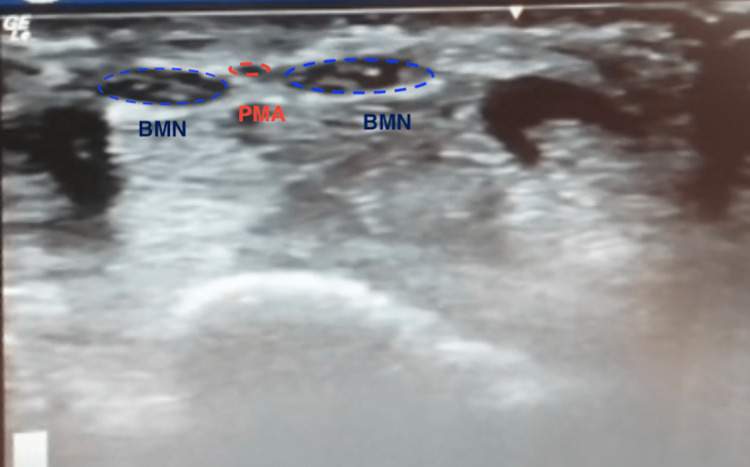
Sonographic imaging of the bifid median nerve and persistent median artery bifid median nerve (BMN), persistent median artery (PMA)

A 38-year-old female patient applied to our clinic with complaints of numbness and pain in her left hand for five months. She had no history of systemic diseases. The physical examination revealed hyperesthesia in the median nerve distribution and a positive Tinel sign. There was no motor deficit, sensory loss, or reflex abnormality of the arm. NCS was performed and normal motor and sensory conduction velocities were reported. An ultrasound of the wrist and median nerve revealed a BMN and persistent median artery (PMA) (Figure 1).

The combined median nerve cross-sectional area (CSA) was 7.94 mm^2^. US-guided 2 ml 5% dextrose hydrodissection was performed (Video 1). The Quick DASH (disability of the arm, shoulder, and hand) score decreased from 25% to 6.8% in the first week.

**Video 1:** 5% dextrose hydrodissection technique of the bifid median nerve The needle was directed from the radial to the ulnar side. 5% dextrose was injected from both between the median nerve and the flexor tendons and between the median nerve and the flexor retinaculum. bifid median nerve (BMN), persistent median artery (PMA)

It was noted that her well-being was maintained at the fourth-month follow-up.

Case 2

A 32-year-old female patient was admitted to the clinic with pain and numbness and a tingling sensation along the median nerve distribution in her left hand. She had no history of systemic diseases. The physical examination revealed positive Tinel and Phalen tests. No abnormality was found by physical and neurologic examination. NCS was performed. Median nerve sensory nerve action potential (SNAP) and compound muscle action potential (CMAP) parameters were reported as normal. BMN was seen on the sonographic evaluation. The combined median nerve CSA was 8.27 mm^2^. Injection of 2 ml dextrose 5% around the median nerve was performed under US guidance. In the first week, the Quick DASH score decreased from 29.5% to 10.8%. The patient reported that discomfort was minimal. A second injection was performed. At the seventh-month follow-up, she was symptom-free.

Case 3

A 28-year-old female patient was admitted to the clinic with dull, aching discomfort and paresthesia in her hands. Her symptoms woke her from sleep and she shook out her hands to try to relieve her symptoms. She had no history of systemic illness. The Phalen sign and nerve compression test revealed positive results. There was no weakness and sensory loss of arm muscles or abnormality of reflexes. NCS was normal. Neuromuscular US showed BMN. The combined CSAs of the right and left median nerve were 8.01 mm^2^ and 7.65 mm^2^, respectively. Two ml of 5% dextrose was injected around the median nerve. At the first week visit following the injection, her symptoms regressed. At the sixth-month follow-up, she was symptom-free.

## Discussion

The diagnosis of CTS is clinical for patients with characteristic symptoms and signs. Electrodiagnostic studies are mostly using screening tools for confirming the diagnosis of CTS and for excluding other conditions in the differential diagnosis. However, mild CTS may not produce any nerve conduction abnormalities. In addition, 10% to 25% of NCS results are falsely negative [1]. Sonographic examination of the carpal tunnel and the median nerve is accurate at diagnosing and is recommended as a supplement to electrodiagnostic studies [4]. It may show morphological changes and increased median nerve CSA [5]. The cut-off values of CSA for CTS diagnosis were ranging from 8.5 to 10 mm^2^ and diagnostic sensitivity and specificity differ in studies [4]. Presented cases had typical CTS symptoms but normal NCS results. Sonographic examination of them showed the BMNs and CSAs of ​​the median nerve close to the lower border for CTS. The relationship between CTS and BMN is controversial. While some studies claim that BMN is an independent risk factor for CTS, it has also been shown that BMN has a similar prevalence in symptomatic and asymptomatic individuals [5,6]. If the BMN is accepted as one of the causes of CTS, this is due to its relatively larger CSA [6].

Corticosteroids are the most commonly used medications for carpal tunnel injections. The mechanism of their action mostly depends on their anti-inflammatory properties. The adverse effects of corticosteroids range from skin discoloration and irritation at the injection site to neurotoxicity, Thenar muscle atrophy, and tendon rupture [7]. Studies have shown that dextrose 5% is not harmful to the nerves even if injected into nerve fascicles [3]. Moreover, dextrose 5% has been reported to have similar efficacy as triamcinolone for improving pain intensity and functional limitations in daily life [8]. Its mechanism of action is thought to depend on hydrodissection and adhesiolysis [9]. In these cases, since the diagnosis was not proven by NCS, so 5% dextrose hydrodissection was chosen so as not to take any potential risk of a glucocorticoid injection. Following the intervention, symptom relief was achieved.

In addition to being a safer method, 5% dextrose injection reduces symptoms more and for longer than other conservative treatment options. In a meta-analysis conducted in 2020 comparing dextrose 5%, platelet-rich plasma, corticosteroid, saline injections, and splinting, it was stated that the best result in symptom relief in the first three months was obtained with dextrose 5% [10]. In another study comparing 5% dextrose and corticosteroid injections in the treatment of mild to moderate CTS, the results of 5% dextrose injection showed greater improvement in pain and function at the fourth and sixth months of follow-up [11]. Similarly, in our cases, well-being continued at four, six, and seven months of follow-up.

Limitations

Three example patients who responded positively to the treatment procedures were selected; therefore, the findings cannot necessarily be generalized to other patients. This case series included a small, homogeneous group of CTS patients who had similar clinical presentations and treatment methods; thus, variants may have resulted in different outcomes.

## Conclusions

BMN was claimed to be an independent risk factor for CTS. Mild CTS may not cause any nerve conduction abnormalities and 10% to 25% of NCS results are falsely negative. In patients with BMN whose diagnosis could not be confirmed with NCS, US-guided 5% dextrose hydrodissection resulted in a safe and significant improvement in paresthesia and pain. Considering that 5% dextrose in the treatment of CTS does not damage the nerves and has a similar efficacy with triamcinolone in improving the quality of daily life, it may be preferred over corticosteroids, especially in cases where the diagnosis is uncertain.
